# Alternative Splicing and Extensive RNA Editing of Human *TPH2* Transcripts

**DOI:** 10.1371/journal.pone.0008956

**Published:** 2010-01-29

**Authors:** Maik Grohmann, Paul Hammer, Maria Walther, Nils Paulmann, Andreas Büttner, Wolfgang Eisenmenger, Thomas C. Baghai, Cornelius Schüle, Rainer Rupprecht, Michael Bader, Brigitta Bondy, Peter Zill, Josef Priller, Diego J. Walther

**Affiliations:** 1 Department of Human Molecular Genetics, Max Planck Institute for Molecular Genetics, Berlin, Germany; 2 Department of Biology, Chemistry, and Pharmacy, Free University Berlin, Berlin, Germany; 3 Neuropsychiatry and Laboratory of Molecular Psychiatry, Charité-Universitätsmedizin, Berlin, Germany; 4 Institute for Legal Medicine, Ludwig Maximilians University, Munich, Germany; 5 Laboratory of Molecular Biology of Peptide Hormones, Max Delbrück Center for Molecular Medicine, Berlin, Germany; 6 Department of Psychiatry, Ludwig Maximilians University, Munich, Germany; Yale University, United States of America

## Abstract

Brain serotonin (5-HT) neurotransmission plays a key role in the regulation of mood and has been implicated in a variety of neuropsychiatric conditions. Tryptophan hydroxylase (TPH) is the rate-limiting enzyme in the biosynthesis of 5-HT. Recently, we discovered a second TPH isoform (TPH2) in vertebrates, including man, which is predominantly expressed in brain, while the previously known TPH isoform (TPH1) is primarly a non-neuronal enzyme. Overwhelming evidence now points to *TPH2* as a candidate gene for 5-HT-related psychiatric disorders. To assess the role of *TPH2* gene variability in the etiology of psychiatric diseases we performed cDNA sequence analysis of *TPH2* transcripts from human *post mortem* amygdala samples obtained from individuals with psychiatric disorders (drug abuse, schizophrenia, suicide) and controls. Here we show that *TPH2* exists in two alternatively spliced variants in the coding region, denoted *TPH2a* and *TPH2b*. Moreover, we found evidence that the pre-mRNAs of both splice variants are dynamically RNA-edited in a mutually exclusive manner. Kinetic studies with cell lines expressing recombinant *TPH2* variants revealed a higher activity of the novel TPH2B protein compared with the previously known TPH2A, whereas RNA editing was shown to inhibit the enzymatic activity of both TPH2 splice variants. Therefore, our results strongly suggest a complex fine-tuning of central nervous system 5-HT biosynthesis by *TPH2* alternative splicing and RNA editing. Finally, we present molecular and large-scale linkage data evidencing that deregulated alternative splicing and RNA editing is involved in the etiology of psychiatric diseases, such as suicidal behaviour.

## Introduction

Serotonin (5-hydroxytryptamine, 5-HT) is a monoaminergic neurotransmitter involved in multiple facets of behavioural control [1], and it has been known for more than four decades that tryptophan hydroxylase (TPH; EC 1.14.16.4) perfoms the first-step and rate-limiting step in its biosynthesis [1], [2]. The serotonergic projection system is the most extensive monoaminergic system in the brain of vertebrates, with its roots in a handful of 5-HT-synthesizing neurons within the midbrain, pons, and medulla oblongata, which altogether constitute the raphe nuclei B1–B9 [1]. These few serotonergic raphe neurons innervate most cortical and subcortical brain areas, including the amygdala, a brain structure critically involved in the modulation of emotional behaviour related to anxiety, fear and reward [3], [4]. Dysregulations in the serotonergic system in the brain have been implicated in a variety of psychiatric disorders, such as depression, suicide, schizophrenia and addiction, which are accompanied by abnormal amygdala function [5].

Recently, a second *TPH* gene (*TPH2*) was identified, which encodes for the main 5-HT-synthesizing enzyme in neurons, whereas the previously known *TPH* gene (*TPH1*) is predominantly expressed in peripheral tissues [1], [6]. While TPH1 is still intensively investigated with regard to its role in developmental processes in embryos and nourishing mothers [7], [8], cancer [9], [10], platelet functions in primary haemostasis [11], liver regeneration [12], insulin secretion [13], and pulmonary hypertension [14], psychiatric 5-HT research now mainly focuses on TPH2 [1], [6], [15], [16], [17], [18], [19]. Although TPH1 has a higher catalytic rate than TPH2 [20] and since in several human brain areas, *TPH1* mRNA is more abundant than *TPH2* [21], TPH1 protein was not detectable by immunohistochemistry [22]. Thus, TPH1 seems not to contribute to brain 5-HT biosynthesis but it cannot be ruled out that impaired TPH1 activity leads to psychiatric illness due to metabolic disorders, such as diabetes [13] or impaired liver function [12].

Numerous studies have identified associations of single nucleotide polymorphisms (SNPs) in the human *TPH2* gene with psychiatric diseases [16], [17], [18], [19]. These studies mainly focused on non-coding SNPs and only few functional data exist. For example, we previously reported the association of a *TPH2* promoter SNP (-614T>A; rs11178997) with reduced transcriptional activity [23], whereas another allelic *TPH2* promoter variant (-844G>T; rs4570625) was shown to associate with amygdala hyperexcitabilty in reaction to emotional stimuli [24], [25]. A functional coding *Tph2* SNP was first described in mouse, where the highly conserved proline^447^ is changed to arginine by the SNP C1473G resulting in a 55% decrease of 5-HT biosynthesis when expressed in PC12 cells [26]. Consistently, BALB/cJ and DBA/2J mouse strains, homozygous for the allele 1473G, showed substantially reduced 5-HT-synthesizing activity in the brain [26]. To date, three non-synonymous *TPH2* SNPs have been associated with psychiatric disorders in humans, which severely impair TPH2 enzymatic activity by causing the amino acid substitutions p.P206S (c.757C>T; exon 6), p.W303R (c.907C>T; exon 7), and p.R441H (c.1322G>A; also known as 1463G>A; exon 11) [27], [28], [29]. Interestingly, we and others tried to confirm the SNP c.1322G>A at the genomic level in more than 5,000 patients of matched collectives without any success [15], [29], [30], [31], [32], [33]. In addition, splice variants in the non-coding 3′-UTR were described, but no functional effects were detected on 5-HT biosynthesis by them [34]. As brain 5-HT biosynthesis is regulated by TPH2 [6], [26], and *TPH2* SNPs increase the risk for psychiatric disorders [27], [28], [29], we decided to further analyze the polymorphic variability of the human *TPH2* gene and focused on its coding region.

Here we show by cDNA sequence analysis of *post mortem* RNA samples obtained from the human amygdala that *TPH2* transcripts exist in at least two alternatively spliced variants in the coding region, namely *TPH2a* and *TPH2b*. Moreover, extensive RNA editing of both *TPH2* isoforms leads to protein variants with distinct catalytic properties. Finally, our data indicate that drug abuse may disturb RNA editing and that imbalanced RNA editing might be involved in the pathogenesis of psychiatric disorders.

## Results and Discussion

### Human *TPH2* Is Alternatively Spliced

**Figure 1:**
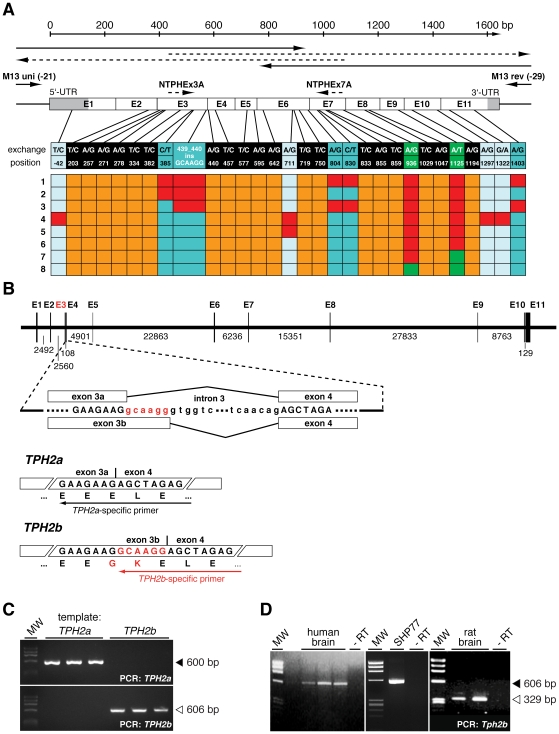
Human *TPH2* exists in two splice variants. (A) Sequencing strategy of *TPH2* cDNA clones obtained from human amygdala of patients with psychopathological disorders and controls. Sequence alignments with the *TPH2* mRNA reference sequence (GenBank NM_173353) led to the identification of 29 SNPs and a 6 bp insertion in exon 3 (n = 104 independent sequences). A compilation of representative *TPH2* cDNA clones (1–8) and the positions of all found SNPs are shown; red boxes indicate the presence of a SNP in the corresponding clone. Eight SNPs were present in dependence of the insertion, forming two mutually exclusive polymorphism patterns. SNPs detectable in presence of the insertion (*TPH2a*) are indicated in dark blue, light blue SNPs were found only in their absence (*TPH2b*). The SNPs at the green positions correspond to the known SNPs *rs7305115* and *rs4290270*. The insertion is a product of alternative splicing of intron 3. (B) Schematic representation of the alternative splicing of *TPH2* pre-mRNA. In higher vertebrates splicing of intron 3 usually occurs at the highly conserved GC splicing donor site (SDS) resulting in the known *TPH2*, now called *TPH2a*. In humans, primates and rats a GT dinucleotide exists 6 bp downstream of the GC SDS, and acts an alternative SDS leading to the inclusion of two additional amino acids, Gly and Lys. This longer *TPH2* isoform is now referred to as *TPH2b*. (C) Specificity of splice-specific *TPH2* primers on plasmid DNA. (D) RT-PCR using splice-specific primers showed the presence of *TPH2b* transcripts in normal human and rat brain and also human neuroendocrine SHP77 cells.

To identify functional SNPs in the human *TPH2* gene, we analyzed *post mortem* brain samples from drug abuse and suicide victims, schizophrenic patients, and controls without a psychiatric history (a full description of the collectives is included in methods). RNA samples were obtained from the amygdala and transcribed into cDNA to amplify, clone and sequence the *TPH2* open reading frame and parts of the untranslated regions. By alignment of the obtained sequences to the *TPH2* mRNA reference sequence (GenBank NM_173353), we detected multiple SNPs in nearly all *TPH2* exons (Figure 1A; Table S1). Among these, only three were known previously, namely the database SNPs *rs7305115* (c.936A>G) and *rs4290270* (c.1125A>T) and the recently reported SNP c.1322G>A (also known as G1463A) [29].

Most notably, we detected a 6 bp insertion (c.439_440insGCAAGG) between exons 3 and 4 by this procedure, which corresponds to a novel splice isoform. Intron 3 starts with a non-canonical GC splicing donor site (SDS), leading to the fusion of exons 3a and 4 (Figure 1B). However, another GT dinucleotide exists 6 bp downstream of the GC SDS, which operates as an alternative SDS, leading to the inclusion of two additional triplets coding for the amino acids Gly and Lys (exon 3b; Figure 1B). We called this novel *TPH2* splice isoform, *TPH2b*, to discern it from the known *TPH2* reference sequence (GenBank NM_173353), which is now referred to as *TPH2a*.

Interestingly, intron 3 of all higher vertebrates starts with a non-canonical GC SDS (Table S2). Exceptions are fishes, which carry a canonical GT SDS at the same position, suggesting evolutionary conservation of this GC-AG intron for at least 450 million years. GC-AG introns occur with a frequency of only about 0.7% in the human genome, but 60% of them are involved in alternative splicing, especially during embryogenesis [35], [36]. Thus, our findings of alternative *TPH2* splicing suggests that this intron might be important for developmental processes, as 5-HT is known to be involved even in pre-neuronal growth regulation [8], [37], [38]. Notably, the alternative GT SDS found in humans is also present in primates and rats, but not in mice (Table S2), excluding the latter as an animal model for the investigation of *TPH2* alternative splicing.

At the protein level, the Gly-Lys insertion (p.146_147insGK) leads to the interruption of a negatively charged stretch of Glu residues by the positive Lys in the hinge structure between the regulatory and catalytic domains (Figure 1B). The hinge region is crucial for substrate accessibility in all aromatic amino acid hydroxylases [2] and it was recently shown that human TPH2 is no exception in this regard [39]. Hence, this structural feature of TPH2B predicted an impact on its hydroxylating activity (see below).

*TPH2b* transcripts can be found in every individual, even without a psychiatric history, using a splice-specific primer (Figure 1B–D). Moreover, *TPH2b* transcripts are also readily detectable in rat brain and human small cell lung carcinoma SHP77 cells, which express *TPH2* (Figure 1D) together with other neuroendocrine markers. Therefore, our data indicate that TPH2B is an isoform that contributes to the brain 5-HT biosynthesis in a hitherto undefined manner.

### *TPH2a* and *TPH2b* Pre-mRNAs Undergo Editing

Most notably, the analysis of *TPH2* gene variability revealed two distinct patterns of synonymous and non-synonymous base exchanges in dependence of the c.439_440insGCAAGG insertion (Figure 1A; Table 1). Thus, the majority of *TPH2a* transcripts are characterized by the SNPs c.-42T>C (5′-UTR; exon 1), c.711A>G (p.R237; exon 6), c.1297A>G (p.R433G; exon 10), and c.1322G>A (p.R441H; exon 11), whereas *TPH2b* transcripts contained four other polymorphisms, namely c.385C>T (p.Q129X; exon 3), c.804A>G (p.K268; exon 6), c.830C>T (p.P277L; exon 7), and c.1403A>G (p.Q648R; exon 11). Both isoform-specific polymorphism patterns appeared almost without exception together with the database SNPs *rs7305115* and *rs4290270* (Figure 1A). Interestingly, the *TPH2b* polymorphism c.385C>T is a nonsense base exchange, which creates a premature stop codon upstream of the catalytic domain of TPH2 [39] and would represent a null mutation with regard to enzymatic activity. This variant would be expected to be a substrate for nonsense-mediated mRNA decay (NMD) [40], [41], but this is obviously not the case given the ease of its detection (Figure 1D).

**Table 1:** Amygdala-specific editing of *TPH2a* transcripts.

*TPH2* Isoform	Genotype *rs4290270*	No. Clones	Editing Position and Percentual Distribution in the Respective Transcripts			
			*1*	*2*	*3*	*4*
Amygdala						
*TPH2a*	A	48	33%	31%	33%	33%
	T	17	0%	0%	0%	0%
*TPH2b*	A	27	78%	96%	96%	96%
	T	-	-	-	-	-
other brain areas^a^						
*TPH2a*	A	8	0%	0%	0%	0%
	T	19	0%	0%	0%	0%
*TPH2b*	A	11	100%	100%	100%	100%
	T	-	-	-	-	-

The percentual distribution of edited positions in *TPH2a/b* transcripts revealed that *TPH2a* is only edited in the amygdala, while *TPH2b* editing is detectable in all investigated brain areas. Note that in presence of SNP *rs4290270* A neither *TPH2a* editing could be observed nor expression of *TPH2b*.

a Cortex, thalamus, hypothalamus, hippocampus, cerebellum, median raphe, pons, and striatum.

The isoform-specific polymorphism patterns were detected in the vast majority of *TPH2a* and *TPH2b* transcripts, but they cannot be explained by the presence of only two *TPH2* alleles. Therefore, our results prompted us to hypothesize posttranscriptional modification of *TPH2* transcripts by RNA editing, a mechanism known to regulate the activity of many neuronal proteins [42], [43], [44], [45].

Posttranscriptional RNA editing of primary transcripts alters genomically encoded sequences and enables multiple transcripts from a single gene, thereby generating proteomic diversity from a limited number of genes [46]. In humans, RNA editing was first described for the apolipoprotein B (APOB) [47], [48], [49]. A cytidine deaminase of the apolipoprotein B mRNA editing enzyme catalytic polypeptide (APOBEC) family converts a cytidine to uridine in the *APOB* primary transcripts by hydrolytic deamination [50]. This C-to-U editing (C>U) of *APOB* transcripts changes a glutamine codon to a premature stop codon in the intestine, giving rise to a functionally important, truncated 48 kDa protein, whereas the non-edited APOB100 is expressed in liver. In mammals, base exchanges from adenosine-to-inosine (A>I) represent the most common RNA editing mechanism, which influences neurotransmission by modulating the functional properties of glutamate receptors [44], serotonin receptors [43] and potassium channels [42]. The best studied example, the 5-HT_2C_ receptor, is dynamically edited at five positions (A to E) in exon V, leading to multiple receptor isoforms, which differ in constitutive activity and intracellular signal transduction efficacy, thereby modulating the strength of 5-HT neurotransmission [43], [51], [52]. A>I editing is catalyzed by deaminases of the adenosine deaminase acting on RNA (ADAR) family and inosine is recognized as guanosine by the translation machinery [46]. Given that RNA editing mediates fine-regulation of central nervous system (CNS) neurotransmission, dysregulations of RNA editing have been associated with brain disorders [53].

**Figure 2:**
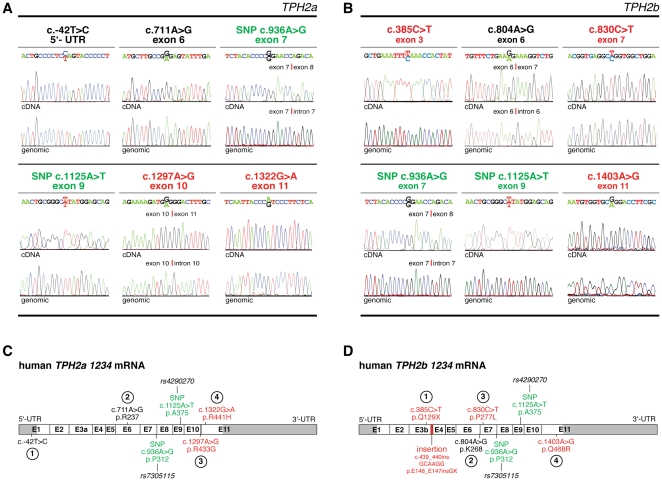
*TPH2a* and *TPH2b* undergo extensive mRNA editing. (A, B) Alignment of genomic *TPH2* sequences and corresponding cDNA traces of *TPH2a* and *TPH2b* transcripts revealed that neither the *TPH2a* SNPs c.-42T>C, c.711A>G, c.1297A>G and c.1322G>A (A) nor the *TPH2b* polymorphisms c.385C>T, c.804A>G, c.830C>T and c.1403A>G are encoded genomically (B), indicating posttranscriptional RNA editing for those positions. Only the SNPs *rs7305115* (c.936A>G) and *rs4290270* (c.1125A>T) could be verified as genuine SNPs at the genomic level. (C, D) The editing patterns for *TPH2a* and *TPH2b* transcripts are mutually exclusive. Four edited positions exist in each alternatively spliced variant, indicated by arabic numbers. Schematic representation of *TPH2a 1234* (C) and *TPH2b 1234* (D) transcripts. Synonymous and non-synonymous base substitutions are indicated in black and red, respectively; SNPs are shown in green.

To assess for *TPH2* editing, we cloned and sequenced the corresponding *TPH2* exons from the genomic DNA of individuals, in whom we detected *TPH2* polymorphisms. However, neither the polymorphisms c.-42T>C, c.711A>G, c.1297A>G and c.1322G>A (*TPH2a*) nor c.385C>T, c.804A>G, c.830C>T and c.1403A>G (*TPH2b*) were found in any of these samples. Only the database SNPs *rs7305115* and *rs4290270* could be detected at the genomic level and were confirmed as genuine SNPs (Figure 2A and 2B). Thus, we found that *TPH2a* and *TPH2b* pre-mRNAs are extensively RNA-edited by a yet unidentified mechanism, which involves mutually exclusive RNA editing patterns (indicated by Arabic numbers) and leads to the expression of the corresponding *TPH2a 1234* and *TPH2b 1234* transcripts, respectively (Figure 2C and 2D). Moreover, detection of partially edited *TPH2a* (*TPH2a 2*, *TPH2a 134*) and *TPH2b* (*TPH2b 1*, *TPH2b 234*) transcripts (Figure 1A) suggests a dynamic *TPH2* RNA editing machinery in analogy to 5-HT_2C_ receptor editing [43]. This results in a wide variety of different TPH2 isoforms, and increases the biochemical diversity and complexity of central 5-HT biosynthesis.

Furthermore, we observed *TPH2a* editing exclusively in the amygdala, whereas *TPH2b* was edited in all brain regions analyzed (Table 1), and also in SHP77 cells (data not shown). Accordingly, our data underscore previous findings that RNA editing is often restricted to discrete brain regions [54].

RNA editing of human *TPH2* transcripts is remarkably miscellaneous (Figure 2) and comprises all editing mechanisms known for mammals, including rare U>C editing [55], which we found for *TPH2a* position 1 (c.-42T>C; 5′-UTR). Interestingly, we also found RNA editing for the c.1322G>A (R441H) polymorphism, which has been associated with major depression [29]. This SNP is currently a matter of major debate, since it could not be confirmed at the genomic level neither in our study (Figure 2A), nor in laboratories worldwide [15], [29], [30], [31], [32], [33]. Thus, its tempting to speculate that the detection of c.1322G>A in blood DNA samples by Zhang *et al*. [29] might be due to a rare *de novo* mutation in elderly patients. It is conceivable that this G>A transition may have resulted from deamination of a methylated cytosine, as c.1322G is part of a CpG dinucleotide, the major target of DNA methyltransferases [56]. The transition c.1322G>A might also have resulted from somatic hypermutation, which was shown to modulate genomic DNA by edited RNA in B cells [57]. However, our results strongly favour RNA editing for c.1322G>A by an extremely rare mechanism, which thus far was only described for the proviral RNA of the human immunodeficiency virus [58]. Thus, *TPH2a* transcripts might offer a unique possibility to study G>A editing in a physiological context. Furthermore, to our knowledge, most mammalian pre-mRNAs are edited by only one mechanism [42], [43], [44], [45]. Thus, *TPH2* transcripts might offer an interesting target for the investigation of how different editing machineries act in concert on a single transcript.

### Editing Defines the Kinetics of TPH2A and TPH2B Isoforms

**Figure 3:**
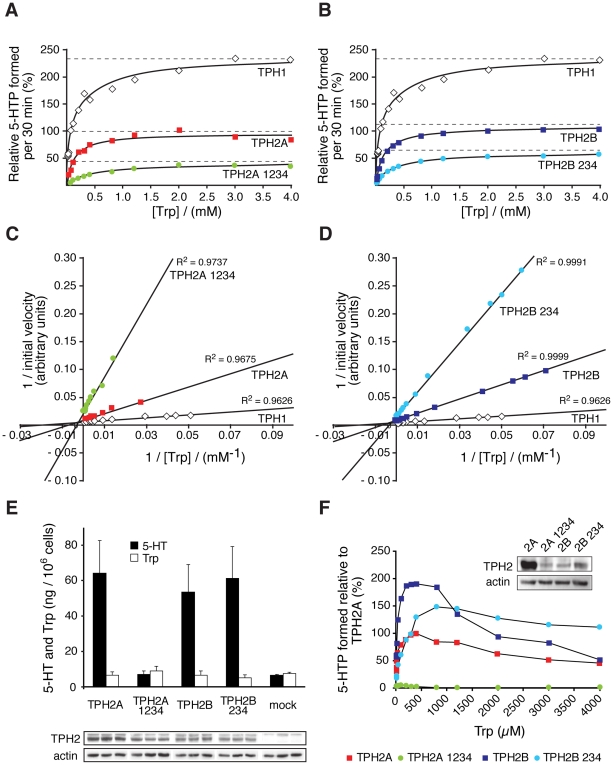
Kinetic properties of TPH2 variants are modulated by RNA editing. (A, B) 5-hydroxytryptophan (5-HTP) formation of TPH2 containing cellular PC12 lysates in presence of the synthetic cofactor 6-methyl-tetrahydrobiopterin (6MPH4). (C, D) Double reciprocal Lineweaver-Burk plots for *K_m_*(W) determination of TPH2 variants. Enzymatic activities of TPH2A and TPH2B, and TPH2A 1234 and TPH2B 234 isoforms were similar, respectively, when using the synthetic cofactor 6MPH4. TPH1 served as a control. RNA editing decreased enzyme activity in both isoforms. Shown are combined data of 4–7 independent experiments. (E) 5-HT and Trp contents of stably transfected PC12 cells. Western blots of the TPH2 variants were used for normalization of 5-HT levels (n = 10 independent experiments). (F) Enzymatic activity of TPH2 variants expressed in non-neuronal HEK293 cells in presence of the natural cofactor tetrahydrobiopterin (BH4). At concentrations above the physiological Trp range of 30–50 µM, all variants except TPH2B 234, exhibit strong substrate inhibition. Shown are combined data of 6 independent experiments.

Alternative splicing and RNA editing of human *TPH2* transcripts generate multiple protein variants with potentially different properties. To address this question, we stably expressed *TPH2a* and *TPH2b* and their edited isoforms in rat pheochromocytoma PC12 cells and performed kinetic studies with the cellular lysates (Figure 3A–D). Instead of *TPH2b 1234* we analyzed a *TPH2b 234* cDNA, which we also detected in our samples (Figure 1A, Table 1), but lacks nonsense editing at position 1 (c.385C>T; p.Q129X) and enables the expression of a full-length TPH2B 234 protein.

It has been helpful to use synthetic analogues of TPH cofactors to discern catalytic differences between TPH enzymes isolated from different tissues (Table S3), because the differences are rather small if the natural cofactor tetrahydrobiopterin (BH4) is used [2], [20]. Therefore, we used 6-methyl-tetrahydrobiopterin (6MPH4), which provides the highest resolution of kinetic differences, and obtained hyperbolic plots for TPH2A, TPH2B, TPH2A 1234 and TPH2B 234, according to the Michaelis-Menten equation (Figure 3A and 3B). The analysis of kinetics by double-reciprocal Lineweaver-Burk plots allowed determination of the Michaelis constants (*K_m_*) for Trp of the indicated TPH2 variants (Figure 3C and 3D). The *K_m_*(Trp) values for TPH2A and TPH2B were nearly similar (Table 2), but significantly higher for the edited variants, indicating a reduced affinity for the substrate Trp (Table 2). However, *K_m_*(Trp) values for TPH2A and TPH2B were close to the previously reported constants and consistently higher than for TPH1 (Table S3) [20], [39].

**Table 2:** Kinetic constants of TPH2 variants.

	K_m, Trp_ (µM)6MPH4 (300 µM)	K_m, Trp_ (µM)BH4 (300 µM)
TPH2A	126±9 (n = 6)	16±11 (n = 6)
TPH2A 1234	292±97 (n = 7)*	23±3 (n = 6)
TPH2B	124±11 (n = 4)	37±69 (n = 6)#
TPH2B 234	304±77 (n = 7)*	116±18 (n = 6)* ^,^ #

* : p<0.05 versus non-edited;

# : p<0.05 versus all others.

PC12 cells have been used to directly assess 5-HT synthesis of recombinant TPH2 mutants [29]. The expression of the four TPH2 variants in PC12 cells revealed equal 5-HT contents for TPH2A and TPH2B, and, unexpectedly, also for TPH2B 234, for which we had expected lower activity (Figure 3E). However, it was previously shown that PC12 cells can only store limited neurotransmitter amounts in their vesicles [59]. Thus, it is conceivable that the TPH2 activity in these three stable cell lines by far exceeded their vesicular 5-HT storage capacity. Interestingly, TPH2A 1234-expressing cells did not produce significantly elevated 5-HT levels compared to mock-transfected cells, which contained comparable 5-HT amounts of about 18% of the maximal levels detected (Figure 3E). Therefore, our data indicate a major loss of TPH2A 1234 enzymatic activity by RNA editing and support the recently reported 80% reduction of 5-HT synthesis in PC12 cells expressing the TPH2-R441H mutant [29]. This mutant corresponds to a TPH2A 4 protein, but cumulative effects of the remaining editing positions might also contribute to TPH2A 1234 inactivation. However, our results raise the question whether the 20% residual activity of TPH2-R441H might simply reflect a low intrinsic 5-HT synthesis capacity of PC12 cells, either due to a low endogenous TPH expression or the known substrate promiscuity of tyrosine hydroxylase [2], which is highly expressed in these catecholaminergic cells.

To circumvent any above-mentioned artefactual influences of PC12 cells, we determined the *K_m_*(Trp) values of the TPH2 variants also in non-neuronal HEK293 cells using the natural cofactor BH4 (Table 2). As expected, kinetics with BH4 resulted in a lower resolution of the *K_m_*(Trp) values of the four TPH2 variants (Table 2), but revealed major differences in the relative maximal velocities (*V_max_*) of the corresponding enzymes. At physiological Trp concentrations of 30 to 50 µM, all TPH2 variants obeyed the Michaelis-Menten equation, but showed significant substrate inhibition at higher concentrations (Figure 3F). TPH2A 1234 presented the lowest *V_max_* with 5% of TPH2A and confirmed its enzymatic inactivation by RNA editing, as shown by 5-HT measurements (Figure 3E). Interestingly, TPH2B presented the highest *V_max_*, which was twice as high as for TPH2A and well in accordance with the prediction that the GK insertion into the hinge region allows easier access of the substrates to the catalytic core. RNA editing of *TPH2b* resulted in a 50% decrease of *V_max_* of the corresponding TPH2B 234 protein, which exhibited the highest *K_m_*(Trp) value of all variants (Table 2). However, TPH2B 234 activity, which was still consistently higher than for TPH2A, can be totally abolished by RNA editing at position 1 (c.385C>T; p.Q129X), resulting in a premature stop codon upstream of the catalytic domain [39] and the expression of an inactive, truncated TPH2B 1 protein. Thus, TPH2B might be important for a rapid response in amygdala 5-HT synthesis, when 5-HT levels need to be increased. Furthermore, it possesses the possibility to be rapidly switched off by editing of its RNA at the position 1 (c.385C>T).

Although *TPH2b 1* transcripts would be expected for degradation by NMD [40], [41], we detected them easily (Figure 1D). In this respect, *APOB48* transcripts are protected from NMD by the C>U editing machinery, which allows for the expression of the truncated APOB form [47], [48], [49]. Accordingly, our data suggest that *TPH2b 1* transcripts might also be protected from NMD by the same mechanism and point to a physiological role of the TPH2B truncation.

Our data show that the activities of both TPH2 isoforms are inhibited by RNA editing and can even be completely abolished by this mechanism (Figure 3F). Moreover, TPH2 physiologically acts as a tetramer [2], [39] and forms functional heteromers with its mutant variants [27], [29]. Coexpression studies revealed intermediate enzymatic activities of the resulting hybrids as compared with the corresponding wildtype and mutant homotetramers [27], [29]. Therefore, TPH2 proteomic diversity generated by alternative splicing and RNA editing suggests further control of 5-HT biosynthesis at the level of enzyme oligomerization.

In conclusion, our results underscore that human CNS 5-HT biosynthesis is a highly regulated process, which is based on the expression of a wide variety of functional TPH2 proteins with different properties. This should enable a complex fine-tuning of 5-HT biosynthesis in response to agonist stimulation in order to maintain optimal 5-HT neurotransmission.

### *TPH2* Editing Is Abnormal in Individuals with Psychiatric Disorders

Interestingly, *TPH2a 1234* editing (Figure 2C) was found elevated by 20% in transcripts obtained from the amygdala of drug abuse and suicide victims compared to controls (Table S4). Thus, the known 5-HT hypofunction in psychiatric disorders may result at least in part from the expression of the low active TPH2A 1234 protein in these individuals. In contrast, no *TPH2a* editing could be detected in schizophrenic patients (Table S4). Moreover, high levels of *TPH2b* editing were found in all patients and controls, whereas *TPH2b* transcripts of suicides and schizophrenics showed a substantial decrease in editing at position 1 (c.385C>T) by 50% and 30%, respectively (Table S4). Thus, dysregulations in *TPH2* editing could be involved in the pathogenesis of psychiatric diseases or may directly result from substance abuse.

For comparison, altered RNA editing of 5-HT_2C_ receptor transcripts was found in depressed suicides and schizophrenics [60], [61], [62], [63], leading to distinct receptor isoforms with different activities. In rodents, changes in 5-HT_2C_ receptor editing were evident in response to stress [51] and 5-HT availability [60], whereas antidepressants were found to antagonize these changes [51], [52], [64]. This complex fine-tuning of the 5-HT_2C_ receptor sensitivity is considered to be a crucial mechanism to keep receptor activation within an optimal range for information processing in face of changing synaptic input [64]. Our findings suggest that brain 5-HT biosynthesis is also regulated by *TPH2* pre-mRNA editing, which could be affected by drug abuse and environmental factors in analogy to the 5-HT_2C_ receptor. However, our collective of *post mortem* brain samples is small and conclusions have to be drawn with care. Nonetheless, our findings invite large-scale follow up studies.

### SNP *rs4290270* Regulates *TPH2* Splicing and Editing

Most notably, we never detected *TPH2b* transcripts or editing in the presence of the SNP *rs4290270* A (Figure 1; Table 1). This strongly resembles the recently found regulation of 5-HT_2C_ receptor splicing by the small nucleolar RNA (snoRNA) HBII-52 and its deregulation by mutagenesis of the 5-HT_2C_ receptor mRNA binding site [65]. However, we could not find a complementary snoRNA for the sequence context of the *TPH2 rs4290270* SNP in the existing databases. Nonetheless, such *trans*-acting factors are only one possible explanation, as different expression or splicing efficacies due to *rs4290270*-mediated differences in *TPH2* pre-mRNA secondary structure could be also responsible.

**Figure 4:**
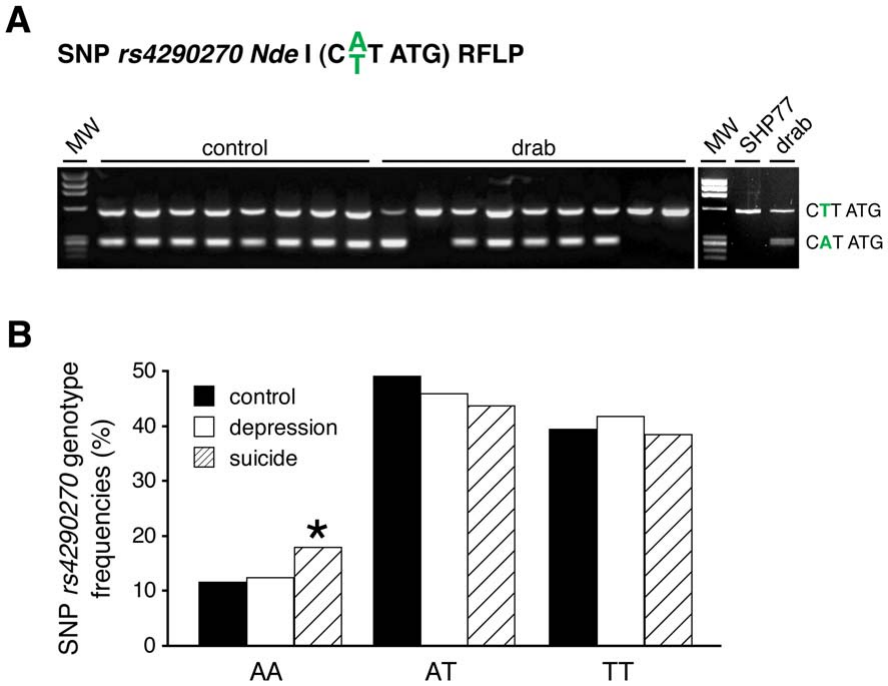
Alternative splicing and editing only occurs in presence of SNP *rs4290270* T. (A) The individual genotype of SNP *rs4290270* can be easily detected by restriction fragment length polymorphism (RFLP) analysis with *Nde* I in drug abusers (drab) and controls. (B) Large scale *rs4290270* genotyping revealed a genetic predisposition for suicidality of homozygous A/A-carriers. The distribution was in the Hardy Weinberg equilibrium. *: p<0.05; controls: n = 373; major depression: n = 436; suicide: n = 369.

Fortunately, *rs4290270* is part of the palindromic recognition sequence of *Nde* I, thus individual genotypes can be easily determined by restriction fragment length polymorphism (Figure 4A). In line with the finding that SHP77 cells exhibit alternative *TPH2* splicing and editing of *TPH2b*, these cells are homozygous for *rs4290270* T (Figure 4A). Thus, these cells may represent a suitable cell culture system to investigate the dynamics of splicing and editing.

Importantly, we detected a significantly higher frequency of the A/A genotype of the SNP *rs4290270* in a cohort of 369 suicides, as compared with 436 patients with major depression and 373 controls (Figure 4B). Thus, while we still have to elucidate the underlying mechanism of how *rs4290270* A affects *TPH2* alternative splicing and editing, the data demonstrate a genetic predisposition of homozygous A-allele carriers for suicide.

### Implications for Psychiatric Research

Our functional data imply disturbed TPH2 activity in drug abuse, suicide, and schizophrenia. The regulation of TPH2 expression reveals an unprecedented mechanism of mutually exclusive editing of the alternatively spliced isoforms *TPH2a* and *TPH2b*. For this reason, our data establish *TPH2* as an excellent subject for future investigations of the underlying RNA editing machineries.

Recent studies have tried to explain the reduced 5-HT neurotransmission in psychiatric disorders with disturbances in TPH2 expression [66], [67], [68], [69], [70]. However, based on our results that alternative splicing and RNA editing lead to TPH2 variants with different kinetic properties, we conclude, that neither the currently used RNA-based techniques, such as real time PCR or RNase protection assays, nor immunohistochemical protein methods allow a proper estimation of TPH2 enzymatic activity in psychiatric research. Based on the data presented here, careful re-examination of recent reports on TPH2 expression disturbances in neuropsychiatric diseases is mandatory. Moreover, since we are now in knowledge of the alternative splicing and editing that governs TPH2 activity, powerful new methods are eagerly awaited to assess for the editing status of *TPH2* transcripts to gain insight into the regulation of 5-HT synthesis in the human brain.

## Methods

### Ethics Statement

All clinical investigations have been conducted according to the principles expressed in the Declaration of Helsinki and approved by the Ethics Committee of the Medical Faculty of the Ludwigs Maximilians University (LMU) Munich (Head: Prof. Dr. Gustav Paungartner, Members: Prof. Dr. Eckhard Held, Prof. Dr. Wolfgang Eisenmenger, PD Dr. Thomas Beinert, Prof. Dr. Hans Ulrich Gallwas, Prof. Dr. Detlef Kunze, Dr. Viktoria Mönch, Prof. Dr. Randolph Penning, Prof. Dr. Klaus Hahn, Prof. Dr. Klaus Jürgen Pfeifer, and Dr. Christian Zach). Ethikantrag, Projekt Nr. 213/00; positive vote from: 12.05.2005 “Genetische, biochemische und funktionelle Untersuchungen an depressiven Patienten und gesunden Kontrollpersonen”. Ethikantrag, Projekt Nr. 164/00; positive vote from: 14.04.2003 “Genetische Polymorphismen bei Suizidenten”. Written informed consent was given by the patients and healthy volunteers. Autopsy samples: The autopsies were court ordered from the state attorney. In that case informed consent from the next of kin is not required, because relatives have no possibility for intervention. Within these autopsies it is necessary to take routinely additional tissue probes for probable further investigations. The probes of the present study originate from these investigations. The Ethics Committee of the LMU Munich approved this procedure. All autopsies, including those of the control individuals were performed according to the legal requirements. They were court ordered according to the German legal situation from the state attorney due to unknown causes of death. For the control individuals the natural cause of death was verified finally by these autopsies. In all of these cases (patients and controls) informed consent from the next of kin is not required, because relatives have no possibility for intervention. Blood and brain samples were exclusively taken during the routine autopsies to perform the court ordered analysis. Furthermore *post mortem* material will be preserved for subsequently necessary investigations on behalf of the state attorney. Blood and brain samples were never taken for research. For research projects we use only remaining post mortem samples which have been released and approved for use in research by the Ethics Committee of the LMU Munich. As described, the consent for research use of autopsy tissues will be given by the localEthics Committees of the universities. This is the current procedure in legal medicine in Germany.

### Brain Samples

Brain specimens (as indicated in the Ethics statement) were derived from 11 individuals, who died as a consequence of opiate addiction (8 males, 3 females, mean age 30.4±9.8 years; *post mortem* interval (PMI): 14.5±9.6 hours), 8 suicide victims (6 males, 2 females, mean age 42,8±9.4 years; PMI: 18.8±13.7 hours) and 7 schizophrenic patients (5 males, 2 females, mean age 43±13.5 years; PMI: 23.9±6.4 hours). The control tissues were obtained from 10 individuals, who died suddenly from CNS-unrelated diseases (5 males, 5 females, mean age 44.7±15.8 years; PMI: 19.9±7.6 hours). Causes of death were acute cardiac failure (n = 5), accident (n = 3), and homicide (n = 2). The clinical, respectively medical, data sheets of the control individuals were available and excluded any lifetime psychiatric or neurological disorders. According to the medical records, there was no history of psychopharmacological medication, alcohol or drug abuse. Additionally, a toxicological report for all individuals was provided and negative for additional drug intoxification, whereas information on pre-existing psychiatric disturbances was missing for the suicide victims. All individuals were Caucasians from the same geographical region in southern Germany.

### Molecular Biological Methods

All cloning procedures, PCR (used primers are indicated in Table S5), and immunoblotting were conducted according to standard protocols or manufacture's instructions. *Post mortem* brain samples were collected using the RNAlater kit (Qiagen, Hilden, Germany) and immediately frozen at −80°C until RNA extraction. After homogenization, total RNA was extracted from tissues using the RNeasy Lipid Tissue Midi Kit (Qiagen), treated with DNase I (Invitrogen, Carlsbad, CA, USA) and dissolved in RNase-free water. cDNA was synthesized from 2 µg RNA using MMLV reverse transcriptase and random hexamer primers (Invitrogen). The *TPH2* coding sequence was amplified with ORF-fw and ORF-rev primers and subcloned into pCR®-XL-TOPO (Invitrogen). The obtained clones were sequenced and aligned with the *TPH2* mRNA reference sequence (GenBank NM_173353). *TPH2* polymorphisms were analyzed genomically by amplification of the *TPH2* exons from genomic DNA using intronic primers (Table S5), subcloning into pGEM®-T easy (Promega, Madison, WI, USA), and DNA sequencing.

Detection of *TPH2* splice isoforms was performed using the TPH2SPL_fw forward primer together with the splice-specific TPH2a_rev and TPH2b_rev reverse primers and the following PCR conditions: 15 s denaturation at 95°C, 10 s annealing at 75°C/70°C, and 30 s elongation at 75°C/72°C for *TPH2a*/*TPH2b*, respectively. The detection of *Tph2b* in the rat brain was carried out using the primers rTPH2ex2A (forward) and TPH2SPLrat (reverse).

*TPH2* expression constructs were generated from *TPH2a/b* cDNAs obtained from patients by reamplification with 6xHisTph2-fw (forward) primer containing an ATG with a Kozak consensus sequence and a 6xHis tag and supsequent cloning into pTargeT™ (Promega). Stable PC12 and HEK293 cell lines were obtained with linearized *TPH2* constructs and DreamFect (OZ Biosciences, Marseille, France), followed by selection of transfected cells with 500 µg mL^−1^ for at least two weeks. Cell cultures were maintained under standard conditions in DMEM supplemented with 10% fetal bovine serum (FBS, HEK293) and 15% FBS/2.5% donor horse serum (PC12) and antibiotics.

### TPH Activity Assay

The activity of cell homogenates was determined as described [6], [8], monitoring for 5-hydroxytryptophan (5-HTP) accumulation by HPLC in presence of the aromatic amino acid decarboxylase inhibitor 3-hydroxybenzylhydrazine hydrochloride (NSD1015). All reagents were purchased from Sigma-Aldrich (St. Louis, MO, USA). In brief, cells were harvested by scraping and washed twice with phosphate-buffered saline, resuspended in 75 mM tris-acetate buffer (pH 7.5), and lyzed by sonication. After withdrawal of an aliquot for protein determination, the homogenates were immediately preincubated in 100 µL buffer containing 2 mg/mL catalase, 25 mM DTT and 100 µM Fe(NH_4_)_2_(SO_4_)_2_ for 10 min at 30°C in the dark. The pre-incubated samples were incubated at 37°C for 30 min after addition of 400 µL 15 mM tris-acetate buffer (pH 6.4) containing the indicated concentrations of L-Trp, 300 µM 6-methyl-tetrahydrobiopterin (6MPH4) or tetrahydrobiopterin (BH4) and 2 mM NSD1015. The reaction was terminated by addition of 300 mM perchloric acid (final concentration) and centrifugation for deproteination. The cleared supernatants were directly analyzed using reverse phase HPLC with fluorometric detection (HPLC-FD) as previously described [8]. The measured 5-HTP levels were normalized to the amount of TPH2 protein in each lysate by immunodetection using mouse anti-WH3 (TPH2, Sigma-Aldrich) and goat anti-actin (Santa Cruz Biotechnology, Santa Cruz, CA, USA) antibodies.

### 5-HT Measurement

To determine 5-HT levels in stable *TPH2*-expressing PC12 cell lines, 1.7 million cells were homogenized in 100 µL buffer containing 5 mM sodium metabisulfite and 300 mM perchloric acid (Sigma-Aldrich). Cleared supernatants were directly used for HPLC-FD measurement as previously described [8]. Cell pellets were boiled in 100 µL SDS loading buffer for 5-HT normalization to the TPH2 protein expression levels by immunoblotting.

### Subjects for Genotyping

The case sample of suicide victims consisted of 369 individuals (269 males, 100 females; mean age: 46.42 years±17.77 years). Of these, 290 committed violent suicides, as e.g. hanging (36%), shooting (18%), penetrating lesions (7%), jumping from height, drowning and lying under a train (17%). 79 employed soft suicide methods, such as intoxication with drugs or other substances (21%). Blood samples for DNA extraction were obtained in the course of autopsy at the Institute for Legal Medicine of the LMU Munich. There was no information on pre-existing psychiatric disturbances.

A total of 436 unrelated Caucasian patients with major depression (270 males, 166 females; mean age: 48.69±14.07 years), hospitalized in the Psychiatric Department of the LMU Munich and diagnosed according to the DSM-IV and ICD-10 criteria were included in the study. All patients were interviewed by experienced psychiatrists using the Structured Clinical Interview for DSM-IV disorders (SCID-I). Severity of depression was assessed using the 17-item Hamilton Rating Scale for Depression (HAMD-17) and the Clinical Global Impression Scale (CGI). Only subjects with a minimum score of 18 on the HAMD-17 scale were included in the study. Patients with severe organic disorders were excluded to avoid cases with secondary depression. Furthermore, all patients with comorbidity of other psychiatric disturbances (e.g. substance/alcohol dependence, personality disorders, anxiety disorders) were excluded. The patient sample contained significantly more females than males as compared with the control sample (62%/38% versus 48%/52%; p = 0.001, χ^2^ = 11.3, df = 1). Because of no significant differences concerning all other investigated variables (age, clinical variables such as CGI and HAMD-17 scores), males and females were not analyzed separately.

As control group, 373 ethnically matched subjects were selected from the general population (185 males, 188 females; mean age: 44.42±16.00 years). All probands were screened for psychiatric disturbances using personality questionnaires (MMPI, NEO-FI, TCI) and a short structured interview with a psychiatrist. Probands with known history of psychiatric disorders were excluded from the study. All patients and controls were of Caucasian origin from the German population and came from the same geographical area in southern Germany. Blood was collected from these subjects for DNA extraction; patients and controls participated after giving written informed consent. The study was approved by the ethics committee of the Medical Faculty of the LMU Munich (project number 213/00; positive vote from: 12.05.2005).

### Genotyping of SNP *rs4290270*

Genomic DNA was isolated from whole blood according standard procedures. The SNP *rs4290270* was genotyped applying the TaqMan® technology (Assay-on-Demand; assay-ID: C_26385365) on an ABI7000 system (Applied Biosystems, Foster City, CA, USA). The standard PCR reaction was carried out using TaqMan® Universal PCR Master Mix reagent kit according to the manufacture's instructions.

### Statistics

All data are presented as means ± SEM and p-values are from two-tailed Student's t-tests type 3. Genotype frequencies were tested for Hardy-Weinberg equilibrium as described [71]. Values of p<0.05 were considered as statistically significant.

## Supporting Information

Table S1 Identified SNPs in the human *TPH2* gene. The positions of base exchanges are indicated according the *TPH2* mRNA reference sequence (GenBank NM_173353) and the described nomenclature system.
(0.07 MB DOC)

Table S2 Exon-intron boundaries of *Tph2* genes of higher vertebrates. The consensus sequence of the five species is indicated by ‘cons’. Alternative 3′-SDS in rats giving rise to four *rTPH2* isoforms.
(0.05 MB DOC)

Table S3 Compiled kinetic constants for mammalian TPH1/2 isoforms.
(0.05 MB DOC)

Table S4 *TPH2a* and *TPH2b* editing in the amygdala of the tested individuals with psychiatric disorders.
(0.06 MB DOC)

Table S5 *TPH2* primers used for PCR amplification.
(0.05 MB DOC)
