# Supplementary material for: Alternative Splicing and Extensive RNA Editing of Human TPH2 Transcripts
Source: PLoS One. 2010 Jan 29;5(1):e8956. doi: 10.1371/journal.pone.0008956 (PMC2813293; doi:10.1371/journal.pone.0008956)
Supplement: Table S1 — Identified SNPs in the human TPH2 gene. The positions of base exchanges are indicated according the TPH2 mRNA reference sequence (GenBank NM_173353) and the described nomenclature system. (0.07 MB DOC) [file pone.0008956.s001.doc]

**Table S1. Identified SNPs in the human *TPH2* gene.** The positions of base exchanges are indicated according the *TPH2* mRNA reference sequence (GenBank NM_173353) and the described nomenclature system.^1^

| **Position (cDNA)** | **Base exchange** | **SNP-ID** | **Type^a^** | **Amino acid** | **Exon** |
| --- | --- | --- | --- | --- | --- |
|  |  |  |  |  |  |
| 100 | c.-42T>C |  | n.c. | n.c. | 5'-UTR |
| 344 | c.203T>C |  | n.s. | p.F68S | 3 |
| 398 | c.257A>G |  | n.s. | p.E86G | 3 |
| 412 | c.271A>G |  | n.s. | p.M91V | 3 |
| 419 | c.278A>G |  | n.s. | p.H93R | 3 |
| 475 | c.334T>C |  | n.s. | p.C112R | 3 |
| 523 | c.382T>C |  | n.s. | p.F128L | 3 |
| 526 | c.385C>T |  | n.s. | p.Q129X | 3 |
| 581 | c.440A>G |  | n.s. | p.E147G | 4 |
| 598 | c.457T>C |  | n.s. | p.W153R | 4 |
| 718 | c.577T>C |  | n.s. | p.Y193H | 5 |
| 736 | c.595A>G |  | n.s. | p.M199V | 5 |
| 783 | c.642A>G |  | s. | p.E214 | 6 |
| 852 | c.711A>G |  | s. | p.R237 | 6 |
| 860 | c.719T>G |  | n.s. | p.L240W | 6 |
| 891 | c.750T>C |  | s. | p.C250 | 6 |
| 945 | c.804A>G |  | s. | p.K268 | 6 |
| 971 | c.830C>T |  | n.s. | p.P277L | 7 |
| 974 | c.833T>C |  | n.s. | p.V278A | 7 |
| 996 | c.855A>G |  | s. | p.R285 | 7 |
| 1000 | c.859T>C |  | n.s. | p.F287L | 7 |
| 1077 | c.936A>G | *rs7305115* | s. | p.P312 | 7 |
| 1170 | c.1029T>C |  | s. | p.S343 | 8 |
| 1188 | c.1047A>G |  | s. | p.E349 | 8 |
| 1266 | c.1125A>T | *rs4290270* | s. | p.A375 | 9 |
| 1335 | c.1194A>G |  | s. | p.K398 | 10 |
| 1438 | c.1297A>G |  | n.s. | p.R433G | 10 |
| 1463 | c.1322G>A |  | n.s. | p.R441H | 11 |
| 1544 | c.1403A>G |  | n.s. | p.Q468R | 11 |

^a^n.c. – non-coding, s. – synonymous SNP, n.s. – non-synonymous SNP. ^1^den Dunnen JT, Antonarakis SE. Nomenclature for the description of human sequence variations. *Hum Genet* 2001; **109:** 121-124.
