# Supplementary material for: Alternative Splicing and Extensive RNA Editing of Human TPH2 Transcripts
Source: PLoS One. 2010 Jan 29;5(1):e8956. doi: 10.1371/journal.pone.0008956 (PMC2813293; doi:10.1371/journal.pone.0008956)
Supplement: Table S2 — Exon-intron boundaries of Tph2 genes of higher vertebrates. The consensus sequence of the five species is indicated by ‘cons’. Alternative 3′-SDS in rats giving rise to four rTPH2 isoforms. (0.05 MB DOC) [file pone.0008956.s002.doc]

**Table S2. Exon-intron boundaries of *Tph2* genes of higher vertebrates.** The consensus sequence of the five species is indicated by ‘cons’. Alternative 3’‑SDS in rats giving rise to four *rTPH2* isoforms.

**Position Species Splicing Acceptor Site Splicing Donor Site**

**Exon 1** human ...CAGCTCAACA gtgagtactacg...

mouse ...CAGCTTAACA gtgagtatcgag...

rat ...CGGCTTAACA gtgagtatggag...

chicken ...CGGCCTCACC gtaagtgacggg...

fish ...ATCCTCCATG gtgagtgctgaa...

**cons ...MDSCYYMAYV gtragtrhbrvr...**

**Exon 2** human ...acattcctgaag CTAAATAAAC...GCTCTTTCAG gtgaatgtgaaa...

mouse ...actttcttgaag CAAAATAAGG...ACTATTCCAG gtaaacacagag...

rat ...actctcttgaag CAAAATAAGG...ACTCTTCCAG gtaaacgcagac...

chicken ...atccatttgtag ATAAACAGAT...GCTCTTCCAG gtaaagctcaca...

fish ...gcagctccgcag GACATCCTTT...GCTCTTTCAG gtcagtgtgcac...

**cons ...ryhbhyyyghag VWMAWYMDDB...RCTMTTYCAG gtvarbvyvvmv...**

**Exon 3** human ...gtgtttccacag GAAAAACGTG...GAGGAAGAAG ***GCAAGGgt***ggtc...

mouse ...atgtttccctag GAAAAACATG...GAGGAAGAAG ***gc***aaggctgatt...

rat ...tgttttccacag GAAAAACACG...GAGGAAGAAG ***GCAAGGgt***ggtt...

chicken ...ttctttcttcag GAGAAGCATG...GATGAGGAAG ***gc***aaggctactt...

fish ...ttgtgtttaaag GAGAAGCATG...GCAGAGACCG gtaagtctgaaa...

**cons ...dkbtktyyhhag GARAARCRYG...GMDGARRMMG gyaagkstrvwh...**

**Exon 4** human ...tgttttcaacag AGCTAGAGGA...TGACCACCCA gtaagtgtccag...

mouse ...tgttacctgcag ATCTCGAGGA...CGACCATCCA gtaagtgtccgt...

rat ...tgtttt***cagcag*** AACTAGAGGA...CGACCACCCA gtaagtgtccgt...

chicken ...tatcttctgcag ATCTTGACTG...AGATCACCCT gtaagtgtaaca...

fish ...tcttctttccag ACTGTTTGGA...TGATCATCCA gtaagttaatat...

**cons ...tvtyhyywvcag AXYKHKWSKR...HGAYCAYCCW gtaagtkwmhvd...**

**Exon 5** human ...tttgtgtttaag GGATTTAAGG...GTTATAAATA gtaagtacctgt...

mouse ...tttctgtttaag GGATTTAAGG...GCTATAAATA gtaagtacctgc...

rat ...tttctgtttaag GGATTCAAGG...GTTATAAATA gtaagtacctgc...

chicken ...tccttgcttaag GGATTTAAAG...GCTATAAATA gtaagtactggt...

fish ...tctcttttgcag GGCTTTAAAG...CAAATTGCAA gtatagattata...

**cons ...tyybtkytkmag GGMTTYAARG...SHWATWRMWA gtawrkayydkh...**

**Exon 6** human ...tctcctgcctag TGGTCAGCCC...TTTCTGAAAG gtaagatttcac...

mouse ...ctgcctgcctag TGGTCAGCCC...TTTCTGAAAG gtaagatcgagc...

rat ...ttgcctgcctag TGGCCAGCCC...TTTCTGAAAG gtaagacctctc...

chicken ...tttttccaccag TGGTCAACCC...TTTCTTAAAG gtaagattcatg...

fish ...tacagtgggcag CCCATCCCAC...TTCTTAAGAG gtaaaacatgtg...

**cons ...yhbhbysvsyag YSSHYMVCMC...TTYYTDARAG gtaarayhbvds...**

**Exon 7** human ...ttgcctttttag AAAGGTCTGG...CCCCAGAACC gtgagtacctac...

mouse ...ttgtctctttag AGCGATCTGG...CCCCGGAACC gtgagtattgca...

rat ...ttgtctctttag AGCGATCTGG...CCCCGGAACC gtgagtatcgct...

chicken ...ccttcacttcag AAAGGTCTGG...CCCCAGAACC gtaagttgcttc...

fish ...gactctttacag AGAGGTCAGG...CACCAGAGCC gtaagtactagc...

**cons ...bhbycwytwyag ARMGRTCWGG...CMCCRGARCC gtragtwbydxh...**

**Exon 8** human ...cttttttgtcag AGACACATGC...ACTAGCCACG gtgagttcattt...

mouse ...tctccctcacag AGATACATGC...ACTAGCCACG gtgagttcattt...

rat ...gtttcctcacag AGACACATGC...ACTGGCCACG gtgagctacccg...

chicken ...ccattcctctag GGATACATGC...ATTAGCCACT gtgagttaaatt...

fish ...cttgtccaccag GGACACCTGC...GCTGGCAACT gtaagtgtgaca...

**cons ...bywbyyyxhyag RGAYACMTGC...RYTRGCMACK gtragykhvhyd...**

**Exon 9** human ...gtttttcttcag TGCTATTTCT...AGAATTAAAG gtatgaagctgt...

mouse ...tcttctttttag TGCTATTTCT...AGAATTGAAG gtatgaagctgt...

rat ...ctttctttttag TGCTATTTCT...AGAATTGAAG gtatgaagctgt...

chicken ...tatattttacag TGCTATTTTT...AGAACTAAAG gtacgttggttg...

fish ...tttttcccctag TGTTATTTCT...AGAGTTAAGG gtaaaagagcct...

**cons ...bhtwyyyyhyag TGYTATTTYT...AGARYTRARG gtahrwdrsyby...**

**Exon 10** human ...tatattttgcag CACGCCCTTT...AAAAGATGAG gtaaactttttt...

mouse ...tatgttttgcag CATGCTCTTT...AAAAGATGAG gtaaacctgctt...

rat ...tatgttttgcag CATGCTCTTT...AGAAGATGAG gtaagcttgctt...

chicken ...tttattttgcag CATGCTCTTT...AAAAGATGAG gtaaactatttc...

fish ...ttgattatgcag CATGCGCTTT...AGAAAATGAG gtaaatgcataa...

**cons ...twkrttwtgcag CAYGCYCTTT...ARAARATGAG gtaarybhdywh...**

**Exon 11** human ...tttattctgcag GGACTTTGCA...

mouse ...tttattctacag GGACTTTGCA...

rat ...tttattctacag GGATTTTGCA...

chicken ...tttttctttcag GGACTTTGCT...

fish ...tcctcttaacag GGAATTTGCT...

**cons ...tyywyyywdcag GGAHTTTGCW...**
