# Supplementary material for: Alternative Splicing and Extensive RNA Editing of Human TPH2 Transcripts
Source: PLoS One. 2010 Jan 29;5(1):e8956. doi: 10.1371/journal.pone.0008956 (PMC2813293; doi:10.1371/journal.pone.0008956)
Supplement: Table S3 — Compiled kinetic constants for mammalian TPH1/2 isoforms. (0.05 MB DOC) [file pone.0008956.s003.doc]

**Table S3. Compiled kinetic constants for mammalian TPH1/2 isoforms.**

| **Source** | **DMPH4** | **6MPH4** | **BH4** | **Reference** |
| --- | --- | --- | --- | --- |
| ***K_m_ for Trp*** |  |  |  |  |
| rat mesencephalic tegmentum (TPH2) | 248 µM | 108 µM | 14 µM | 1 |
| rat brain stem (TPH2) | n.d. | 125 µM | n.d. | 2 |
| recombinant murine TPH2 | 249 µM | 145 µM | 8 µM | our assays |
|  |  |  |  |  |
| mouse mastocytoma cell line |  |  |  |  |
| P815 (TPH1) | 34 µM | 40 µM | 20 µM | 1 |
| P815 (TPH1) | n.d. | 45 µM | n.d. | 3 |
| P815 (TPH1) | 55 µM | 28 µM | 11 µM | our assays |

^1^Kuhn DM, Meyer MA, Lovenberg W. Comparisons of tryptophan hydroxylase from a malignant murine mast cell tumor and rat mesencephalic tegmentum. *Arch Biochem Biophys* 1980; **199:** 355-361. ^2^Nakata H, Fujisawa H. Purification and properties of tryptophan 5-monooxygenase from rat brain-stem. *Eur J Biochem* 1982; **122:** 41-47. ^3^Nakata H, Fujisawa H. Tryptophan 5-monooxygenase from mouse mastocytoma P815. A simple purification and general properties. *Eur J Biochem* 1982; **124:** 595-601.
