# Supplementary material for: Alternative Splicing and Extensive RNA Editing of Human TPH2 Transcripts
Source: PLoS One. 2010 Jan 29;5(1):e8956. doi: 10.1371/journal.pone.0008956 (PMC2813293; doi:10.1371/journal.pone.0008956)
Supplement: Table S4 — TPH2a and TPH2b editing in the amygdala of the tested individuals with psychiatric disorders. (0.06 MB DOC) [file pone.0008956.s004.doc]

**Table S4. *TPH2a* and *TPH2b* editing in the amygdala of the tested individuals with psychiatric disorders.**

| *TPH2* Isoform | Genotype **rs4290270** | | No. Clones | **Editing Position andPercentual Distribution in the Respective Transcripts** | | | |
| --- | --- | --- | --- | --- | --- | --- | --- |
|  | |  |  | *1* | *2* | *3* | *4* |
|  | |  |  |  |  |  |  |
| **controls** | |  |  |  |  |  |  |
| *TPH2a* | | A | 15 | 13% | 20% | 13% | 13% |
|  | | T | 3 | 0% | 0% | 0% | 0% |
|  | |  |  |  |  |  |  |
| *TPH2b* | | A | 8 | 100% | 93% | 93% | 93% |
|  | | T | - | - | - | - | - |
| **drug abuse** | | |  |  |  |  |  |
| *TPH2a* | | A | 18 | 33% | 33% | 33% | 33% |
|  | | T | 2 | 0% | 0% | 0% | 0% |
|  | |  |  |  |  |  |  |
| *TPH2b* | | A | 4 | 100% | 100% | 100% | 100% |
|  | | T | - | - | - | - | - |
| **suicide** | |  |  |  |  |  |  |
| *TPH2a* | | A | 13 | 38% | 23% | 31% | 31% |
|  | | T | 4 | 0% | 0% | 0% | 0% |
|  | |  |  |  |  |  |  |
| *TPH2b* | | A | 4 | 50% | 100% | 100% | 100% |
|  | | T | - | - | - | - | - |
| **schizophrenia** | | |  |  |  |  |  |
| *TPH2a* | | A | 2 | 0% | 0% | 0% | 0% |
|  | | T | 8 | 0% | 0% | 0% | 0% |
|  | |  |  |  |  |  |  |
| *TPH2b* | | A | 11 | 71% | 91% | 91% | 91% |
|  | | T | - | - | - | - | - |
