# Supplementary material for: Alternative Splicing and Extensive RNA Editing of Human TPH2 Transcripts
Source: PLoS One. 2010 Jan 29;5(1):e8956. doi: 10.1371/journal.pone.0008956 (PMC2813293; doi:10.1371/journal.pone.0008956)
Supplement: Table S5 — TPH2 primers used for PCR amplification. (0.05 MB DOC) [file pone.0008956.s005.doc]

**Table S5. *TPH2* primers used for PCR amplification.**

| **Name** | **Position** | **5’-3’ Sequence** |
| --- | --- | --- |
|  |  |  |
| ORF_fw | 5’‑UTR | CATTGCTCTTCAGCACCAGGGTTCTGG |
| ORF_rev | 3’‑UTR | TGACATTGACTGAACTGCTGCTAAGC |
| 6xHisTph2-fw | 5’-UTR | GCCGCCACCATGCATCACCATCACCATCACATGCAG CCAGCAATGATGATGTTTTCC |
| TPH2ins2_fw | intron 2 | GATTGTCTCTTCCTCTTGTGTGG |
| TPH2ins4_rev | intron 4 | CACACAGGAAACAGCTATGACATACCATCCTTCTAACCCCGTTCC |
| TPH2ins5_fw | intron 5 | GATCCTTTCAGACGCTCATGTGC |
| TPH2ins6_rev | intron 6 | CACACAGGAAACAGCTATGACGAGTCCCTTGCTCTGCTTCC |
| TPH2ins6_fw | intron 6 | TATGTCACTCAGTTGTCAAGAGG |
| TPH2ins7_rev | intron 7 | CACACAGGAAACAGCTATGACCAAGCAGATGGCTCAGTGTTGG |
| TPH2ins8_fw | intron 8 | GGCTTCTGAATTCAGGAAGCG |
| TPH2ins9_rev | intron 9 | CACACAGGAAACAGCTATGACTGCTCATCCATTTGAGTCACACC |
| TPH2ins9_fw | intron 9 | AGTTTCCAATTTACCCTGCACAC |
| TPH2ins11_rev | 3’‑UTR | CACACAGGAAACAGCTATGACAATTGCATGCTTATTAGCCAAGCC |
| NTPHEx3A | exon 3 | GAATTCAATGAGCTCATCCAGTTGC |
| NTPH2Ex7A | exon 7 | GTACTGGGTGCAGTGGAATACTCTGTAGG |
| TPH2a_rev | exon 3a | CCAGGGCACATCCTCTAGCTCTTCTTC |
| TPH2b_rev | exon 3b | CCAGGGCACATCCTCTAGCTCCTTGC |
| rTPH2ex2A | rat exon2 | CTAGCAAAAGCGAGGACAAGAGAAGCG |
| TPH2SPLrat | rTph2b | CCACGGCACATCCTCTAGTTCCTTGC |
